# Cardiac Sympathetic Denervation Suppresses Atrial Fibrillation and Blood Pressure in a Chronic Intermittent Hypoxia Rat Model of Obstructive Sleep Apnea

**DOI:** 10.1161/JAHA.118.010254

**Published:** 2019-02-13

**Authors:** Xuechao Yang, Linfei Zhang, Huan Liu, Yongfeng Shao, Shijiang Zhang

**Affiliations:** ^1^ Department of Cardiothoracic Surgery The First Affiliated Hospital of Nanjing Medical University Nanjing Jiangsu People's Republic of China

**Keywords:** atrial fibrillation, cardiac sympathetic denervation, chronic intermittent hypoxia, sleep apnea, sympathetic nerve activity, Atrial Fibrillation, Electrophysiology, Animal Models of Human Disease, Autonomic Nervous System

## Abstract

**Background:**

Chronic intermittent hypoxia (CIH) is a distinct pathological mechanism of obstructive sleep apnea (OSA), which is recognized as an independent risk factor for cardiovascular diseases. The aims of this study were to ascertain whether CIH induces atrial fibrillation (AF), to determine whether cardiac sympathetic denervation (CSD) can prevent it and suppress blood pressure, and to explore the potential molecular mechanisms involved.

**Methods and Results:**

Sixty Sprague‐Dawley male rats were randomly divided into 4 groups: sham, CSD, CIH, CIH+CSD. The rats were exposed either to CIH 8 hours daily or normoxia for 6 weeks. Cardiac pathology and structure were analyzed by hematoxylin and eosin staining and echocardiogram. ECG, blood pressure, body weight, and blood gas were recorded. Connexin 43 and tyrosine hydroxylase were detected by western blot, immunohistochemistry, and immunofluorescence. CIH induced atrial remodeling, and increased AF inducibility. CSD treatment reduced postapneic blood pressure rises and AF susceptibility, which could attenuate CIH‐associated structural atrial arrhythmogenic remodeling. In addition, CIH‐induced sympathetic nerve hyperinnervation and CSD treatment reduced sympathetic innervation, which may affect CIH‐induced AF‐associated sympathovagal imbalance. Connexin 43 was specifically downregulated in CIH, whereas CSD treatment increased its expression.

**Conclusions:**

These results suggested CIH induces atrial remodeling, increases AF inducibility, results in sympathetic nerve hyperinnervation, and decreases connexin 43 expression, but CSD treatment reduces AF susceptibility, postapneic blood pressure increase, sympathetic innervation, and the alteration of Cx43, which may be a key point in the genesis of CIH‐induced AF.

**Clinical Perspective**

**What Is New?**

- To the best of our knowledge, our study is the first to investigate the potential interplay among chronic intermittent hypoxia, atrial fibrillation, and cardiac sympathetic denervation in animal models.
- Chronic intermittent hypoxia ‐induced atrial remodeling, increased atrial fibrillation inducibility, and led to sympathetic nerve hyperinnervation with decreased connexin 43 expression; cardiac sympathetic denervation attenuated these changes.

**What Are the Clinical Implications?**

- Our study suggests that cardiac sympathetic denervation may have a role in the treatment of some obstructive sleep apnea patients with atrial fibrillation.

## Introduction

Obstructive sleep apnea (OSA), is a common sleep‐related breathing disease, characterized by partial (hypopnea) or complete (apnea) occlusion of the upper airway during sleep and linked to excessive daytime sleepiness.^1^, ^2^ Apnea and hypopnea for >5 hours a day during sleep is defined as OSA, which is classified as mild apnea/hypopnea index (AHI 5–15), moderate (AHI 16–30), or severe (AHI >30).^3^ Symptoms of OSA patients include fatigue, snoring, and daytime sleepiness. However, many patients present without symptoms.^4^ Overnight polysomnography is the gold standard for diagnosing OSA, which is based on AHI to record the number of apnea and hypopnea events per hour.^4^ The high prevalence of OSA, affects 17% of women and 34% of men and is largely undiagnosed and is a modifiable cardiovascular disease risk factor.^5^ Patients with OSA have poor sleep quality, which can result in depression, reduced alertness, and increased risk of motor vehicle accidents.^6^ Using an experimental animal model in the basic study of OSA may contribute to a better understanding of its pathophysiological mechanisms and cardiovascular consequences.^7^ The most frequently used model is chronic intermittent hypoxia (CIH), which repeats hypoxia events during the sleep cycle by exposing experimental animals to the hypoxia/reoxygenation component of OSA. Since the first description of CIH animal model in the early 1990s by Fletcher et al,^8^ it has been extensively used in the research of OSA.^9^, ^10^, ^11^ In addition, CIH is a distinct pathological mechanism of OSA, which is recognized as an independent risk factor for cardiovascular diseases.^10^ In this study, we mimicked OSA by a CIH rat model that presents the characteristics of OSA.

A growing body of evidence has emerged linking the existence and severity of OSA to a higher incidence and prevalence of heart diseases such as atrial fibrillation (AF), systemic hypertension, coronary heart disease, stroke, and heart failure.^5^ It was found that the incidence of AF in OSA patients is 3 times higher than in the general population.^12^ There are an estimated 33 million individuals with AF worldwide, with ≈5 million new cases annually. It is estimated that a quarter of people will develop AF by age 40 years and that AF will affect 5 to 15 million people by 2050.^13^ Autonomic nerve activity has a critical role in the initiation and maintenance of AF, and adjusting autonomic nerve function may help AF control.^14^ Recently, various studies have demonstrated that there are significant nerve sprouting and sympathetic hyperinnervation in sustained AF.^15^

To date, treatment of AF has not been satisfactory. Potential therapeutic treatments for AF include renal sympathetic denervation, ganglionated plexus ablation, novel drug approaches, cervical vagal nerve stimulation, cutaneous stimulation, baroreflex stimulation, and biological therapies.^14^ Clinical and experimental studies have suggested that renal sympathetic denervation has potential anti‐remodeling and atrial antiarrhythmic consequences, which may be associated with inhibition of cardiac sympathetic activity (SA) and atrial electrophysiology.^16^, ^17^ Previous studies have revealed that renal sympathetic denervation can decrease local renal SA, and whole body SA, especially cardiac SA, which is now diffusely used to treat resistant hypertension and AF.^16^ These results indicated that adjuvant sympathetic modulation has a positive effect on AF, opening the way for another notion. However, the impact of cardiac sympathetic denervation (CSD) on AF inducibility and blood pressure (BP) during and after obstructive events is unknown.

The aim of our study was to (1) describe CIH‐induced atrial remodeling, (2) analyze the AF inducibility after CIH, (3) determine whether CSD has the potential to reduce AF susceptibility and postapneic BP rises after CIH, and (4) explore the potential molecular mechanisms involved.

## Materials and Methods

The data that support the findings of this study are available from the corresponding author upon reasonable request.

### CIH Model of OSA

The protocol of this study was approved by the *Jiangsu Province Animal Care* ethics committee, and performed according to the *Animals Care and Use Committee of the Nanjing Medical University*. Sixty Sprague‐Dawley male rats (initial weight 200±20 g; 9 weeks old; from the Laboratory Animal Center of Nanjing Medical University) were selected as a model for this study and were housed in individual cages in a temperature‐controlled (22–24°C) room with a 12‐hour light‐dark cycle. Water and food were available ad libitum in the cages. A total of 60 rats were randomly divided into the sham group, the CSD group, the CIH group, and the CIH+CSD group.

### CSD Treatment

One week after their delivery to the laboratory, a right thoracotomy at the fourth intercostal space and the right CSD were performed on the rats in the CIH+CSD and CSD groups. The sympathetic chain behind the parietal pleura was identified and the stellate ganglion, as well as the thoracic ganglia at Thoracic vertebra T 2 to T 4, and their connections to the spinal cord and cardiopulmonary nerves were transected, and then removed. The rats in the other 2 groups underwent the sham operations. The rats were unconscious during all operations.

### CIH Exposure

CIH and CIH+CSD rats were exposed to an intermittent hypoxia chamber with cycling changes of the hypoxic conditions (2 minutes) 8 hours a day (from 8 am to 4 pm) for 6 weeks. The oxygen analyzer automatically continuously measured the oxygen concentration in these chambers and controlled it through a computer system connected to the gas valve outlet. In each cycle, oxygen concentration was decreased to 6% for the first 30 seconds and maintained for 50 seconds, then increased to 21% for 40 seconds.

### Tissue Preparation

At the end of study, all rats were scanned on a VisualsonicsVevo 2100 ultrasound system (VisualSonics, Canada) to evaluate cardiac function. BPs were measured indirectly by tail arteries in awake rats using a BP‐2000 blood pressure analysis system. Blood gas values were analyzed using a blood gas analyzer in the cardiovascular surgery unit of the First Affiliated Hospital of Nanjing Medical University. The body weight (BW) and heart weight (HW) were evaluated for each rat. Eight hearts from each group were immersed in 4% paraformaldehyde for histological detection, and another 7 hearts were flash‐frozen in −80°C refrigerator.

### Electrophysiological Study

Continuous ECG data were recorded and analyzed in Labchart Pro system (USA). Heart rate (HR), PR interval, P amplitude, P duration, QRS interval, QTc, QT interval, and T amplitude were calculated in lead II. To assess atrial tachyarrhythmia inducibility, 25‐Hz burst pacing (pulse width 2 ms, 4×threshold current) was applied for 3 seconds. Each of the 6 3 seconds burst cycles was spaced 1 second apart. AF was defined as a rapid (>800 beats/min) irregular atrial rhythm, and AF inducibility was defined as AF lasting for at least 1 second immediately after the 6‐burst cycle protocol. If AF was induced after <6 burst pacing cycles, burst pacing was suspended to avoid interfering with the evolution of the AF.

### Hematoxylin and Eosin Staining

The cardiac tissues were immersed in 10% formalin, fastened in paraffin, sectioned at 7 μm. Whole hearts of rats were stained with hematoxylin and eosin (HE), and all photographs were assessed blindly and collected directly by a pathologist.

### Western Blot Analysis

Western blotting was performed as our previous studies,^18^, ^19^ with the antibodies for connexin 43 (Cx43) (anti‐mouse, 1:800; Sigma), or GAPDH (Glyceraldehyde‐3‐phosphate dehydrogenase, anti‐rabbit, 1:1000; Sigma).

### Immunohistochemistry

According to the research method published in our previous articles,^19^, ^20^ the heart tissues from each group were examined by immunohistochemistry with Cx43 antibody (anti‐mouse, 1:100; Sigma), or tyrosine hydroxylase antibody (anti‐rat, 1:200; Sigma).

### Double Immunofluorescent Staining

Double immunofluorescent staining, which was described in our previous research,^18^, ^20^ was used to investigate the heart tissues with the Cx43 antibody (Sigma, 1:50), α‐actinin antibody (a marker of heart, Sigma, 1:100), and β‐catenin antibody (intercalated disk associated protein, Sigma, 1:150).

### Statistical Analysis

All data were analyzed with SPSS 16.0 or GraphPad Prism. All values are the means±SEM. One‐way analysis of variance (ANOVA) followed by Tukey's post hoc multiple comparison tests were used for the statistical analysis. A *P* value of <0.05 was considered statistically significant. Each experiment consisted of at least 3 replicates per condition.

## Results

### BW, HW, and the Ratio of HW/BW

**Figure 1:**
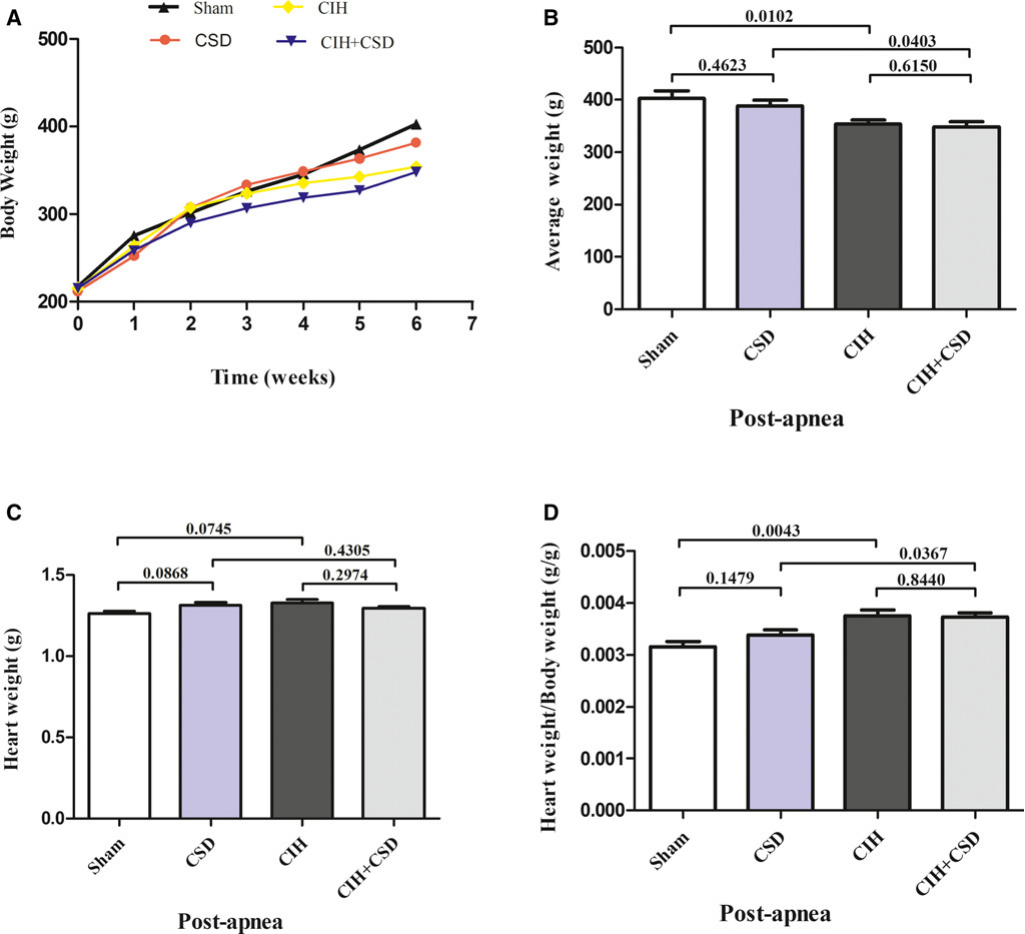
**A**, BW was significantly increased in every group at the end of study; **B**, Average postapnea BW of the CIH and CIH+CSD rats were reduced compared with the sham and CSD rats. **C**, No significant differences of HW among the 4 groups were found. **D**, HW/BW ratio was increased in the CIH and CIH+CSD rats compared with the sham and CSD rats post apnea. BW indicates body weight; CIH, chronic intermittent hypoxia group; CIH+CSD, chronic intermittent hypoxia with cardiac sympathetic denervation group; CSD, cardiac sympathetic denervation group; HW, heart weight; Postapnea, at the end of study; Sham, sham group.

The BW, the whole HW, and the ratio of HW/BW of the rats are shown in Table 1. Initial BW in each group was about (200±20) g, and increased significantly at the end of the study (Figure 1A). However, the postapnea BW of CIH was reduced compared with the sham group (*P*=0.0102) (Table 1, Figure 1B). Compared with the CSD group, the postapnea BW was lower in the CIH+CSD group (*P*=0.0403) (Table 1, Figure 1B). There were no significant differences in HW among the 4 groups. However, analysis of HW showed an increased HW/BW ratio in the CIH and CIH+CSD rats compared with sham and CSD rats (*P*=0.0043, 0.0367) (Table 1, Figure 1D) at the termination of the experiment.

**Table 1:** BW, HW, HW/BW Ratio in Various Groups

Groups	Average Preapnea Weight (g)	Average Postapnea Weight (g)	Heart Weight (g)	Heart Weight/Body Weight (g/g)
Sham	217.3±0.9	402.8±25.5	1.26±0.02	0.0032±0.00017
CSD	215.4±3.3	388.1±19.8	1.31±0.03	0.0033±0.00016
CIH	216.1±4.3	354.1±15.7*	1.33±0.05	0.0038±0.00023*
CIH+ CSD	215.9±0.9	347.9±18.1†	1.29±0.02	0.0037±0.00014†

All values are means±SEM. BW indicates body weight; CIH, chronic intermittent hypoxia group; CIH+CSD, chronic intermittent hypoxia with cardiac sympathetic denervation group; CSD, cardiac sympathetic denervation group; HW, heart weight; Sham, sham group.

*P<0.01 vs sham.

†*P*<0.01 vs CSD.

### Effects of CIH and CSD on Postapneic BP

Postapneic changes in BP are presented in Table 2. The systolic BP was significantly higher in both CIH and CIH+CSD rats, compared with the sham and CSD rats (*P*<0.0001, 0.00314). However, systolic BP in the CIH+CSD rats was considerably decreased compared with the CIH rats (*P*<0.0001), which indicated that CSD abolished postapneic BP surge. Similarly, the diastolic BP was significantly higher in both the CIH (*P*<0.0001 versus sham rats) and CIH+CSD rats (*P*<0.0305 versus CSD rats). Compared with the CIH group, the diastolic BP was lower in the CIH+CSD group (*P*=0.0009) (Table 2). Figure S1 presents detailed information about BP.

**Table 2:** Blood Pressure at Postapnea

	Sham	CSD	CIH	CIH+CSD
Systolic BP, mm Hg	119.6±10.4	116.6±8.9	135.3±12.4*	121.6±6.5†, ‡
Diastolic BP, mm Hg	58.5±6.7	56.9±6.9	70.7±11.6*	61.7±7.55†, ‡

All values are means±SEM. BP indicates blood pressure; CIH, chronic intermittent hypoxia group; CIH+CSD, chronic intermittent hypoxia with cardiac sympathetic denervation group; CSD, cardiac sympathetic denervation group; Sham, sham group.

*P<0.001 vs sham.

†*P*<0.01 vs CSD.

‡*P*<0.001 vs CIH.

### Arterial Blood Gases Values

Blood was gathered at baseline and during the conditioning procedure. Arterial blood gases at baseline did not differ among groups (data not shown). Arterial blood gases during the conditioning procedure identified significant hypoventilation and oxygen desaturation in CIH and CIH+CSD rats (Table 3). The blood pO_2_ of rats in the sham and CSD groups (21% O_2_) was maintained between 96 and 101 mmHg. In the CIH and CIH+CSD group (6% O_2_), the lowest blood oxygen pO2 of rats was maintained between 50 and 59 mmHg. There was significantly reduced PH in the CIH exposure group compared with the sham and CSD groups. No significant differences in Na^+^, K^+^, Ca^2+^, and BE among 4 groups were detected. The statistical values of PH, pCO_2_, pO_2_, and SO_2_ are shown in Figure S2.

**Table 3:** Arterial Blood Gases During Obstructive Apnea

	Sham	CSD	CIH	CIH+CSD
PH	7.40±0.02	7.41±0.03	7.24±0.02†	7.26±0.03§
pCO_2_, mmHg	45.51±2.52	44.54±1.57	65.33±2.62†	64.25±2.95§
pO_2_, mmHg	97.52±2.51	96.50±1.79	55.67±2.49†	52.75±1.48§
SO_2_, %	97.50±1.47	98.52±1.56	43.33±1.25†	43.25±2.86§
Na^+^, mmol/L	139.52±1.53	138.53±1.25	138.66±1.48	139.58±1.39
K^+^, mmol/L	3.75±0.15	3.85±0.05	3.67±0.23	3.83±0.32
Ca^2+^, mmol/L	1.23±0.02	1.24±0.02	1. 29±0.06	1.23±0.04
BE, mmol/L	4.13±0.16	4.14±0.45	4.16±0.17	4.13±0.39
HCO_3_^−^, mmol/L	27.85±0.15	27.05±0.85	30.83±0.81*	29.33±0.39‡

All values are means±SEM. CIH indicates chronic intermittent hypoxia group; CIH+CSD, chronic intermittent hypoxia with cardiac sympathetic denervation group; CSD, cardiac sympathetic denervation group; Sham, sham group.

*P<0.01, ^†^ *P*<0.001 vs sham.

^‡^ *P*<0.01, ^§^ *P*<0.001 vs CSD.

### CIH‐Induced Cardiac Injuries

**Figure 2:**
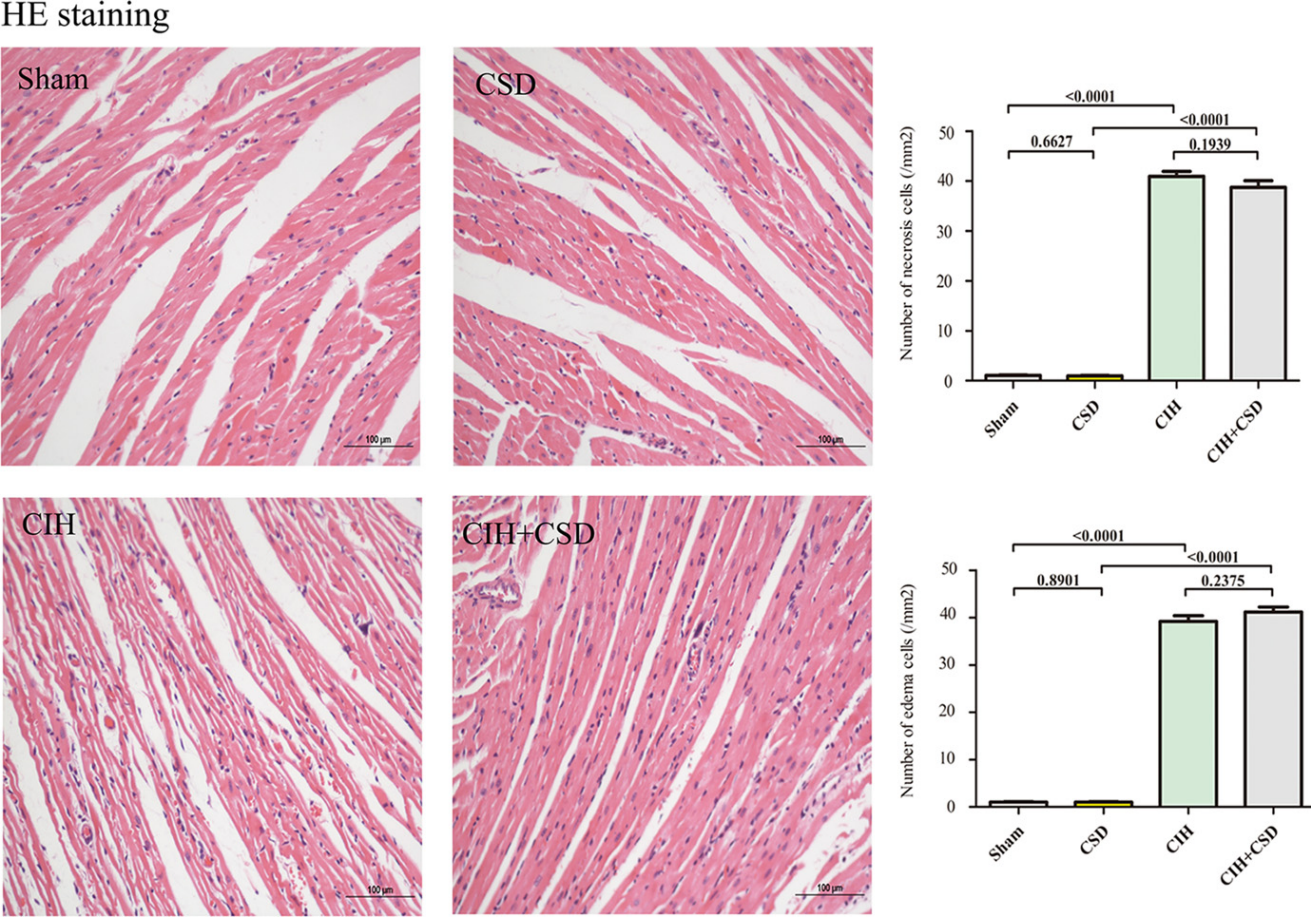
Light microscopic photographs of heart slice (original magnification ×20). HE staining showed that cardiomyocytes were disarrayed with some edema and necrosis, and cardiac structural integrity was compromised with bending cardiac fibers in CIH rats. CIH indicates chronic intermittent hypoxia; CIH+CSD, chronic intermittent hypoxia with cardiac sympathetic denervation group; HE, hematoxylin and eosin staining; Sham, sham group.

To examine the impacts of CIH on rats, HE staining was used to examine the cardiomyocytes. Representative HE staining of left ventricle samples are shown in Figure 2. Cardiomyocytes in the sham and CSD groups were arranged regularly, while the cardiomyocytes in the CIH exposure group were disarrayed with some necrosis and edema. Remarkable increases in necrosis were observed in both CIH (*P*<0.0001 versus sham) and CIH+CSD (*P*<0.0001 versus CSD) groups compared with the sham and CSD groups, respectively. Similarly, edema in both CIH (*P*<0.0001 versus sham) and CIH+CSD (*P*<0.0001 versus CSD) had significant differences. In addition, the cardiac structure integrity was compromised. There were no significant differences of cardiomyocytes between the CIH group and CIH+CSD group. These results indicated that CIH induced cardiac injuries.

### Echocardiographic Parameters

**Figure 3:**
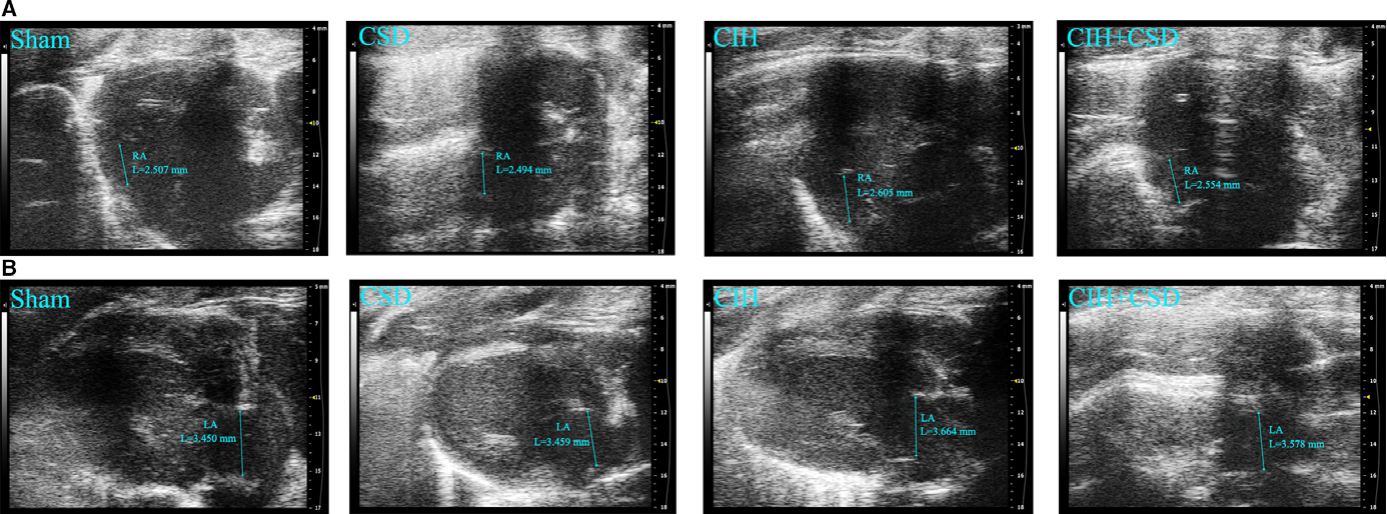
Ultrasonography shows the images of the RAD and LAD. **A**, CIH produced statistically significant RAD dilation, and CSD treatment significantly decreased the RAD dilation. **B**, LAD was significantly increased after CIH, and LAD was reduced by CSD treatment. CIH indicates chronic intermittent hypoxia; CIH+CSD, chronic intermittent hypoxia with cardiac sympathetic denervation group; CSD, cardiac sympathetic denervation; LAD, left atrial diameter; RAD, right atrial diameter; Sham, sham group.

**Figure 4:**
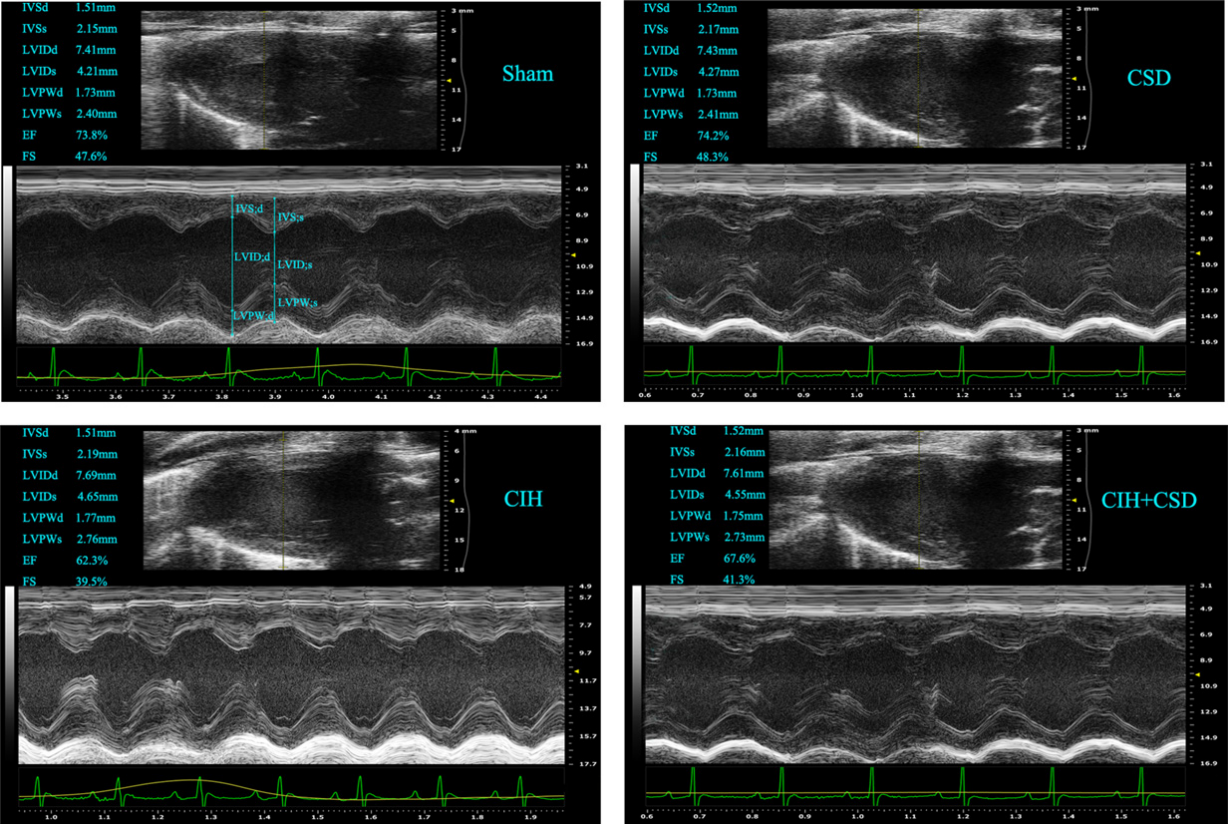
Ultrasonography shows the images of the 4 groups. CIH caused LV hypertrophy and remodeling. EF and FS were significantly decreased after CIH exposure, but CSD treatment increased the FS and EF. CIH indicates chronic intermittent hypoxia; CIH+CSD, chronic intermittent hypoxia with cardiac sympathetic denervation group; CSD, cardiac sympathetic denervation; EF, ejection fraction; FS, fractional shortening; IVSd, the intervernicular septum in diastole; IVSs, the intervernicular septum in systole; LV, left ventricle; LVIDd, left ventricular internal diameter in diastole; LVIDs, left ventricular internal diameter in systole; LVPWd, the left ventricle posterior wall in diastole; LVPWs, the left ventricle posterior wall in systole.

To examine the cardiac function of rats after a 6‐week intermittent hypoxia treatment, we performed echocardiography to study dynamic changes. Table 4 shows echocardiographic parameters at the end study measured in 4 groups. Figure 3 shows images across the left atrial diameter. Left atrial diameter was significantly increased after CIH exposure, which was not observed in the other non‐CIH exposure groups. Correspondingly, CIH produced statistically significant right atrial diameter dilation (Figure 3). Echocardiography (Table 4) showed significant increases in left ventricular internal diameter in systole, left ventricular posterior wall in systole, left ventricular internal diameter in diastole, left ventricular posterior wall in diastole, and left ventricular mass, indicating left ventricular hypertrophy in the CIH exposure rats. However, fractional shortening and ejection fraction were both decreased significantly in CIH exposure rats (Table 4, Figure 4). Interestingly, CSD treatment increased ejection fraction, fractional shortening, and decreased left ventricular internal diameter in systole, left ventricular internal diameter in diastole, left ventricular posterior wall in systole, left ventricular posterior wall in diastole, and left ventricular mass (Table 4, Figure 4). No significant differences in intervernicular septum in systole, intervernicular septum in diastole, right ventricular internal diameter in diastole, and right ventricular anterior wall thickness were found. Figure S3 presents detailed information about echocardiographic parameters among 4 groups. Taken together these results clearly demonstrated that CIH caused cardiac dysfunction and left atrial (LA), right atrial (RA) dilation, while CSD treatment protected cardiac function, and reduced LA, RA dilation.

**Table 4:** Echocardiographic Parameters of the 4 Groups Post Apnea

Parameters	Sham	CSD	CIH	CIH+CSD
LAD, mm	3.44±0.14	3.46±0.01	3.67±0.08†	3.57±0.05∥ ^,^ #
RAD, mm	2.50±0.01	2.49±0.01	2.59±0.02‡	2.56±0.02¶ ^,^ #
IVSd, mm	1.51±0.01	1.52±0.00	1.51±0.05	1.52±0.02
IVSs, mm	2.16±0.11	2.22±0.02	2.19±0.15	2.17±0.10
LVIDd, mm	7.40±0.15	7.43±0.01	7.70±0.23*	7.62±0.12§
LVIDs, mm	4.22±0.20	4.28±0.05	4.66±0.26*	4.59±0.11∥
LVPWd, mm	1.73±0.01	1.73±0.01	1.77±0.01‡	1.76±0.02§
LVPWS, mm	2. 39±0.06	2.43±0.12	2.77±0.27*	2.75±0.21§
RVIDd, mm	2.21±0.08	2.19±0.03	2.18±0.08	2.17±0.10
RVAW, mm	0.52±0.01	0.51±0.01	0.52±0.01	0.52±0.01
EF, %	73.80±1.65	74.40±1.34	62.17±3.48‡	67.55±1.23¶ ^,^ **
FS, %	47.59±2.59	48.45±2.83	39.49±1. 59‡	41.29±1.56∥ ^,^ #
LV mass, mg	847.5±19.4	848.9±12.1	945.2±15.6‡	902.7±18.4¶ ^,^ ††

All values are the means±SEM. CIH indicates chronic intermittent hypoxia group; CIH+CSD, chronic intermittent hypoxia with cardiac sympathetic denervation group; CSD, cardiac sympathetic denervation group; EF, ejection fraction; FS, fractional shortening; IVSd, the intervernicular septum in diastole; IVSs, the intervernicular septum in systole; LAD, left atrial diameter; LV mass, left ventricular mass; LVIDd, left ventricular internal diameter in diastole; LVIDs, left ventricular internal diameter in systole; LVPWd, the left ventricle posterior wall in diastole; LVPWs, the left ventricle posterior wall in systole; RAD, right atrial diameter; RVAW, right ventricular anterior wall thickness; RVIDd, right left ventricular internal diameter in diastole; Sham, sham group.

*P<0.05, ^†^ *P*<0.01, ^‡^ *P*<0.001 vs sham.

^§^ *P*<0.05, ^∥^ *P*<0.01, ^¶^ *P*<0.001 vs CSD.

^#^ *P*<0.05, **P<0.01, ^††^ *P*<0.001 vs CIH.

### ECG Findings

Table 5 shows the ECG findings of 4 groups during sinus rhythm. Remarkable increases in HR were observed in both CIH (*P*<0.0001 versus sham) and CIH+CSD (*P*<0.0001 versus CSD) groups compared with the sham and CSD groups, respectively. Table 5 also showed a decreased HR in CSD (*P*<0.0001 versus sham) and CIH+CSD (*P*<0.0001 versus CIH) groups compared with the sham and CIH groups. P wave duration and T amplitude were increased in both CIH and CIH+CSD groups, whereas differences in PR interval, P amplitude, QRS interval, QT interval, and QTc among 4 groups failed to achieve statistical significance (Table 5). The statistical values of RR interval, HR, P wave duration, and T amplitude are shown in Figure S4. These results indicated that CIH increased postapneic HR, P wave duration, and T amplitude but decreased the RR interval. However, CSD treatment suppressed postapneic HR and P wave duration, and enhanced RR interval.

**Table 5:** ECG Parameters of the 4 Groups Post Apnea

Parameters	Sham	CSD	CIH	CIH+CSD
HR, bpm	337.5±10.9	312.8±9.1*	393.6±16.0†	340.1±8.5‡, §
PR interval, s	0.04978±0.0027	0.04988±0.0028	0.04979±0.0021	0.04985±0.0025
P duration, s	0.01629±0.0015	0.01614±0.0012	0.01921±0.0046†	0.01765±0.0032‡, §
P amplitude, mv	0.12784±0.0079	0.12741±0.0014	0.12760±0.0041	0.12752±0.0059
QRS interval, s	0.01666±0.0009	0.01668±0.0008	0.01666±0.0005	0.01669±0.0005
QT interval, s	0.08041±0.0024	0.08036±0.0025	0.08052±0.0013	0.08053±0.0012
QTc, s	0.17598±0.0026	0.17555±0.0031	0.17588±0.0024	0.17563±0.0019
T amplitude, mv	0.16795±0.0044	0.16799±0.0052	0.19893±0.0052†	0.19905±0.0049‡

All values are means±SEM. CIH indicates chronic intermittent hypoxia group; CIH+CSD, chronic intermittent hypoxia with cardiac sympathetic denervation group; CSD, cardiac sympathetic denervation group; Sham, sham group.

*P<0.001 vs sham.

†*P*<0.001 vs sham.

‡*P*<0.001 vs CSD.

§*P*<0.001 vs CIH.

### AF Susceptibility Changes and Effect of CSD on AF Inducibility

**Figure 5:**
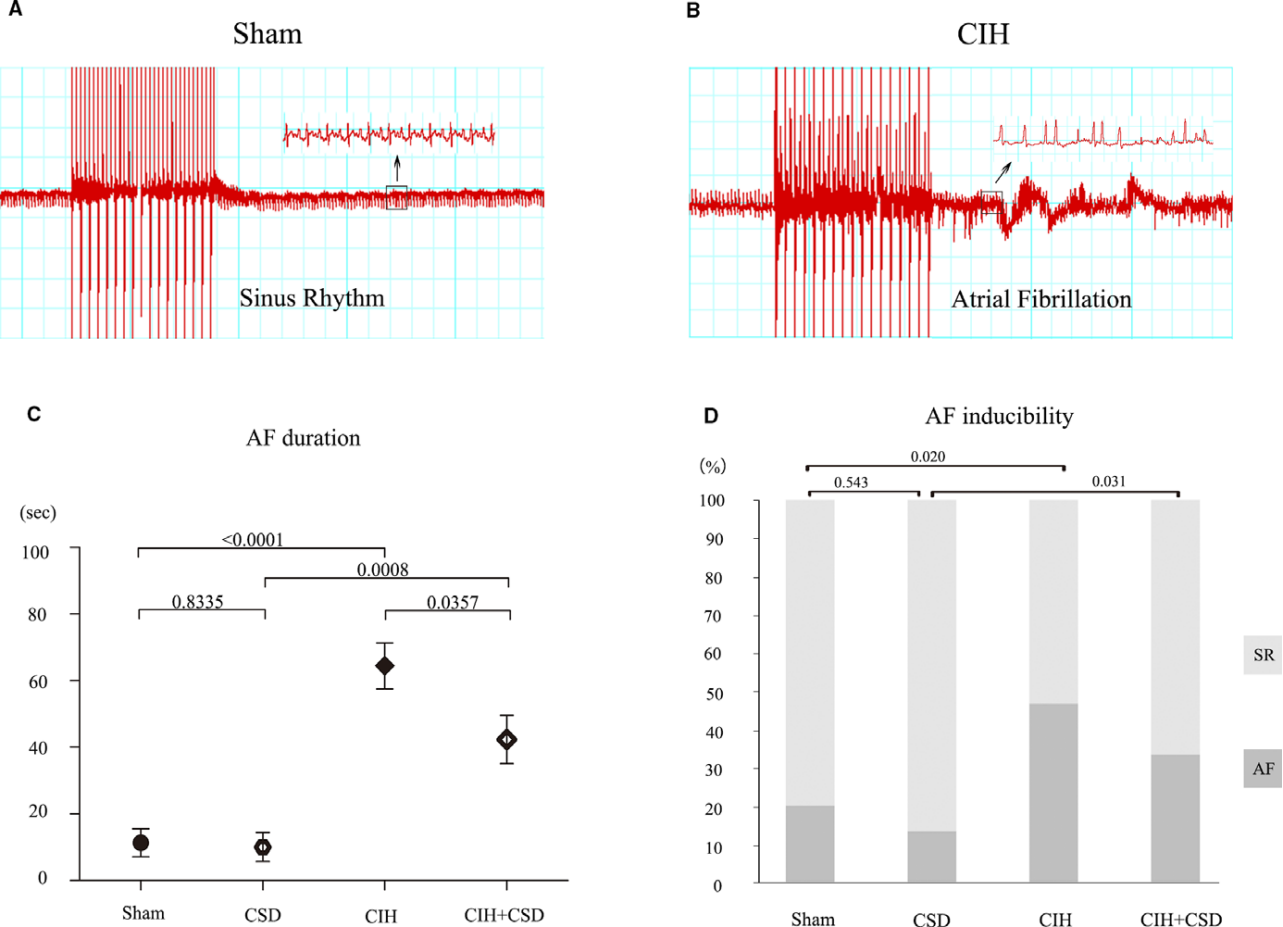
AF susceptibility changes post apnea. Examples of burst pacing‐induced atrial fibrillation (AF) of CIH rats (**B**) and failure to induce AF in a sham rat (**A**). AF duration (**C**) and AF inducibility (**D**) of the 4 groups. AF indicates atrial fibrillation; CIH, chronic intermittent hypoxia; CIH+CSD, chronic intermittent hypoxia with cardiac sympathetic denervation group; CSD, cardiac sympathetic denervation; SR, sinus rhythm.

AF susceptibility changes and the effect of CSD on AF inducibility are shown in Figure 5. Typical burst pacing cycles often succeeded in inducing AF in the CIH exposure rats, while uncommonly inducing AF in the sham and CSD rats. Compared with the sham group, AF duration increased in CIH rats (*P*<0.0001, Figure 5C). Similarly, AF duration in between the CSD and CIH+CSD rats (*P*=0.0008, Figure 5C) had significant differences. AF duration decreased in CIH+CSD rats compared with the CIH group (*P*=0.0357, Figure 5C), indicating that the CSD plays a protective role in AF duration.

We tested AF inducibility in 4 groups. In Figure 5D, the impacts of CSD on AF inducibility are shown. Remarkable increases in AF inducibility were observed in both CIH (*P*=0.020 versus sham) and CIH+CSD (*P*<0.031 versus CSD) groups compared with the sham and CSD groups, respectively. There were no significant differences between sham and CSD group (*P*=0.543). Taken together these results clearly demonstrated that CIH significantly increased AF inducibility in the CIH and CIH+CSD rats.

### The Sympathetic Innervation in RA and LA Among 4 Groups

**Figure 6:**
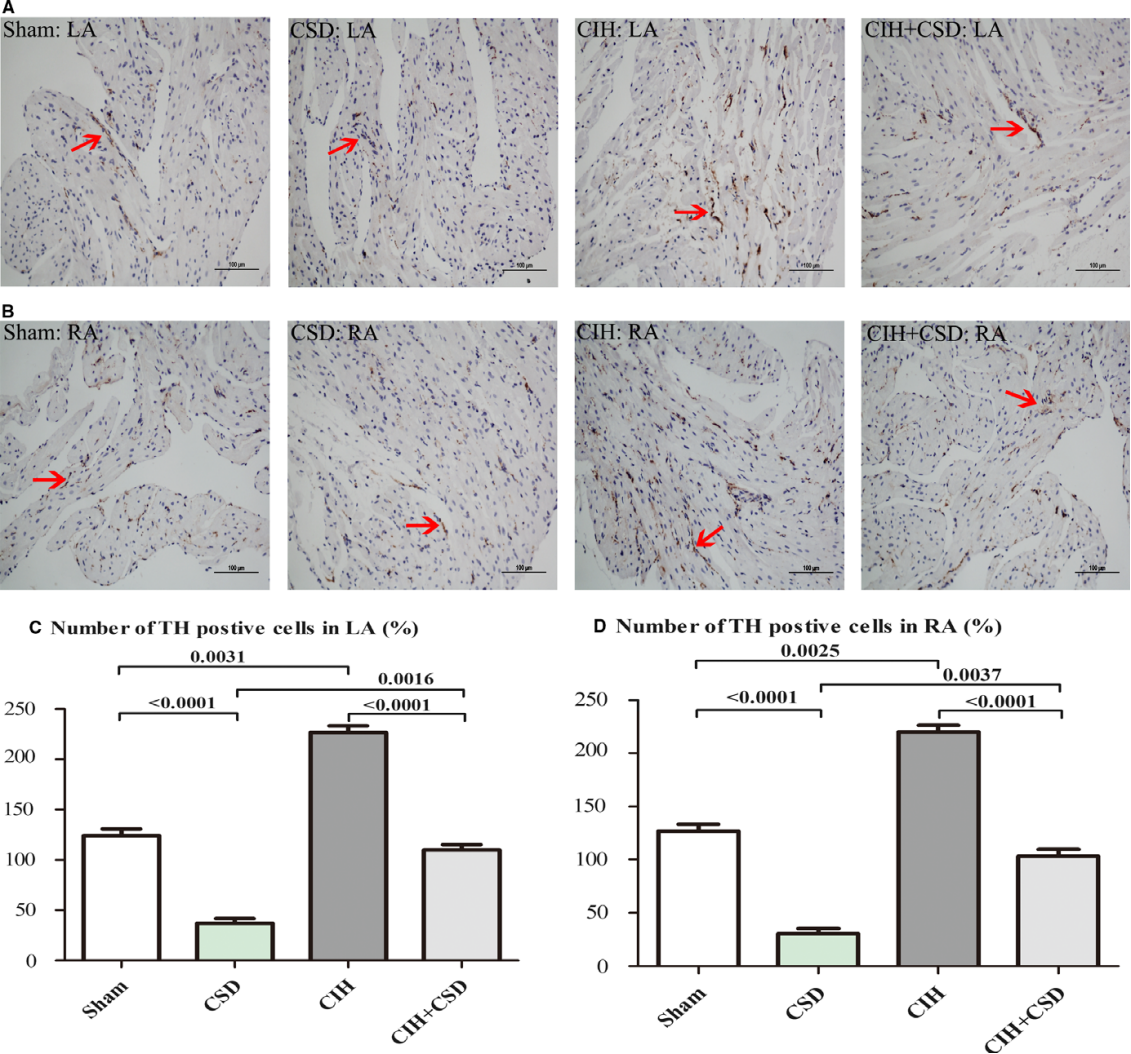
Representative immunohistochemical staining of TH on the LA (**A**) and RA (**B**) among 4 groups. Red arrows indicate tyrosine hydroxylase positive nerves (original magnification ×20). **C**, Quantitative analysis of TH‐positive cell expression in LA of each group. **D**, Quantitative analysis of TH‐positive cell expression in RA of each group. CIH indicates chronic intermittent hypoxia; CIH+CSD, chronic intermittent hypoxia with cardiac sympathetic denervation group; CSD, cardiac sympathetic denervation; LA, left atrial; RA, right atrial; Sham, sham group; TH, tyrosine hydroxylase.

To examine the density of the cardiac sympathetic nerve, immunohistochemical staining was performed on the RA and LA among 4 groups. Significant sympathetic nerve hyperinnervation was examined in the LA of the CIH group compared with the sham group (*P*=0.0031, Figure 6A). Apparently, CSD treatment significantly decreased the density of the tyrosine hydroxylase‐positive nerve in CSD (*P*<0.0001 versus sham) and CIH+CSD (*P*<0.0001 versus CSD) groups. Figure 6B presents images of immunohistochemical staining of tyrosine hydroxylase in RA, which was increased in CIH (*P*=0.0025 versus sham) and reduced in CSD (*P*<0.0001 versus sham) and CIH+CSD (*P*<0.0001 versus CSD) groups. Number of tyrosine hydroxylase‐positive cells were shown in Figure 6C and 6D. Taken together these results clearly indicated that CIH induced sympathetic nerve hyperinnervation and CSD treatment reduced sympathetic innervation.

### Cx43 is Specifically Downregulated After CIH and CSD Treatment Increases Cx43 Expression

**Figure 7:**
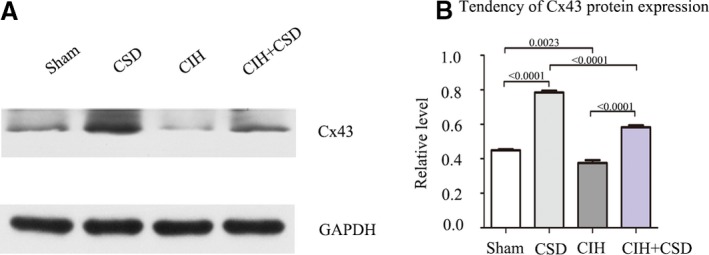
Representative western blot analysis shows the protein expression of Cx43 among 4 groups. **A**, The expression of Cx43 protein was detected by western blots. Cx43 is specifically downregulated after CIH and CSD treatment increases Cx43 expression. GAPDH was used as an internal control. **B**, Quantification of the total Cx43 protein expression of the 4 groups. CIH indicates chronic intermittent hypoxia; CIH+CSD, chronic intermittent hypoxia with cardiac sympathetic denervation group; CSD, cardiac sympathetic denervation; Cx43, connexin 43.

**Figure 8:**
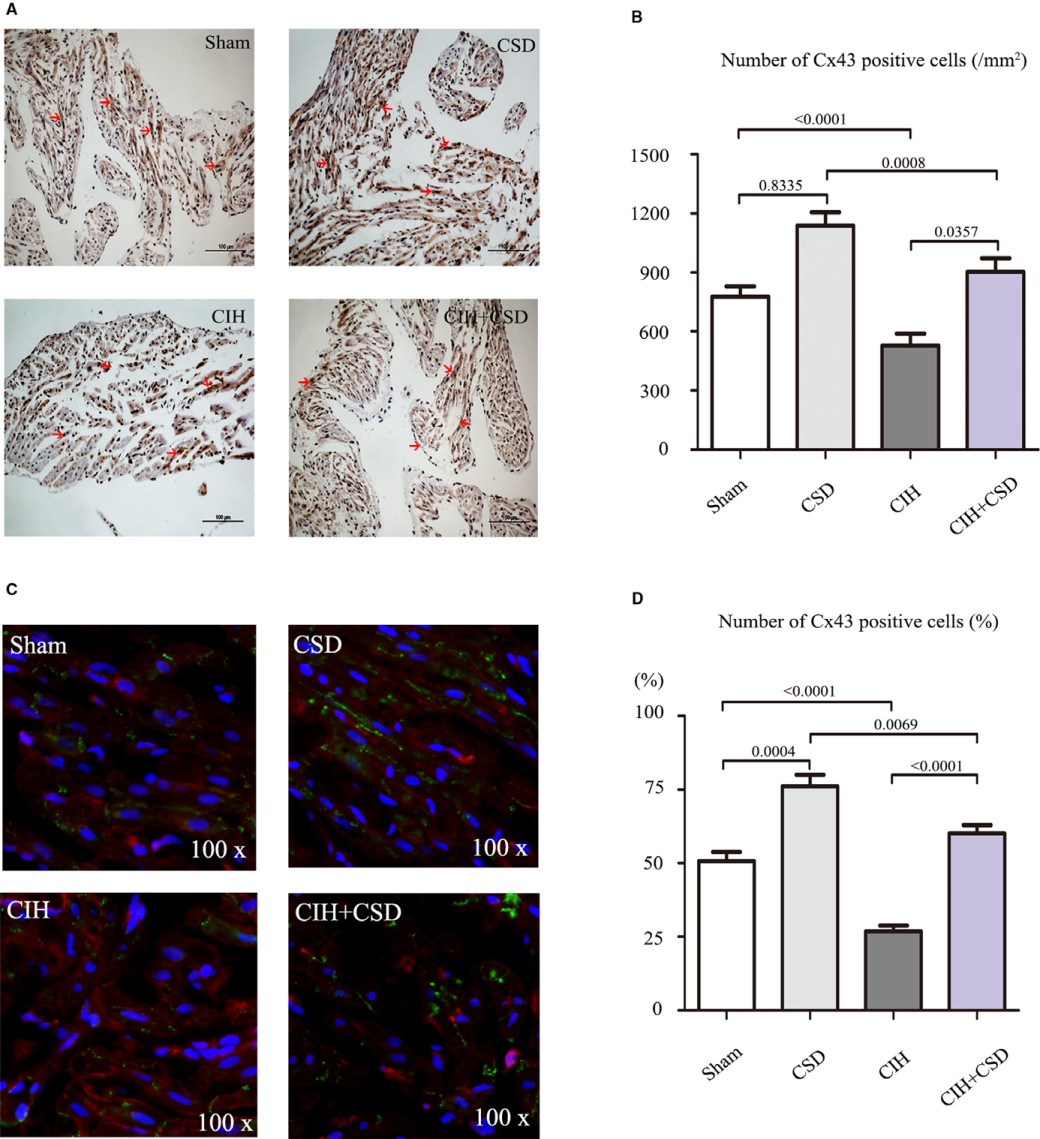
Representative immunohistochemical staining and double immunofluorescent staining of Cx43 of the 4 groups. **A**, Representative immunohistochemical staining of Cx43 of the 4 groups. Red arrows indicate Cx43 expression (original magnification ×20). **B**, Quantitative analysis of Cx43 positive cells of 4 groups. **C**, Examples of Cx43 (green) with α‐actinin (red) staining (original magnification ×100). **D**, Quantitative analysis of cardiomyocyte‐positive cells expressing Cx43 (%) in each group. CIH indicates chronic intermittent hypoxia; CIH+CSD, chronic intermittent hypoxia with cardiac sympathetic denervation group; CSD, cardiac sympathetic denervation; Cx43, connexin 43; Sham, sham group.

**Figure 9:**
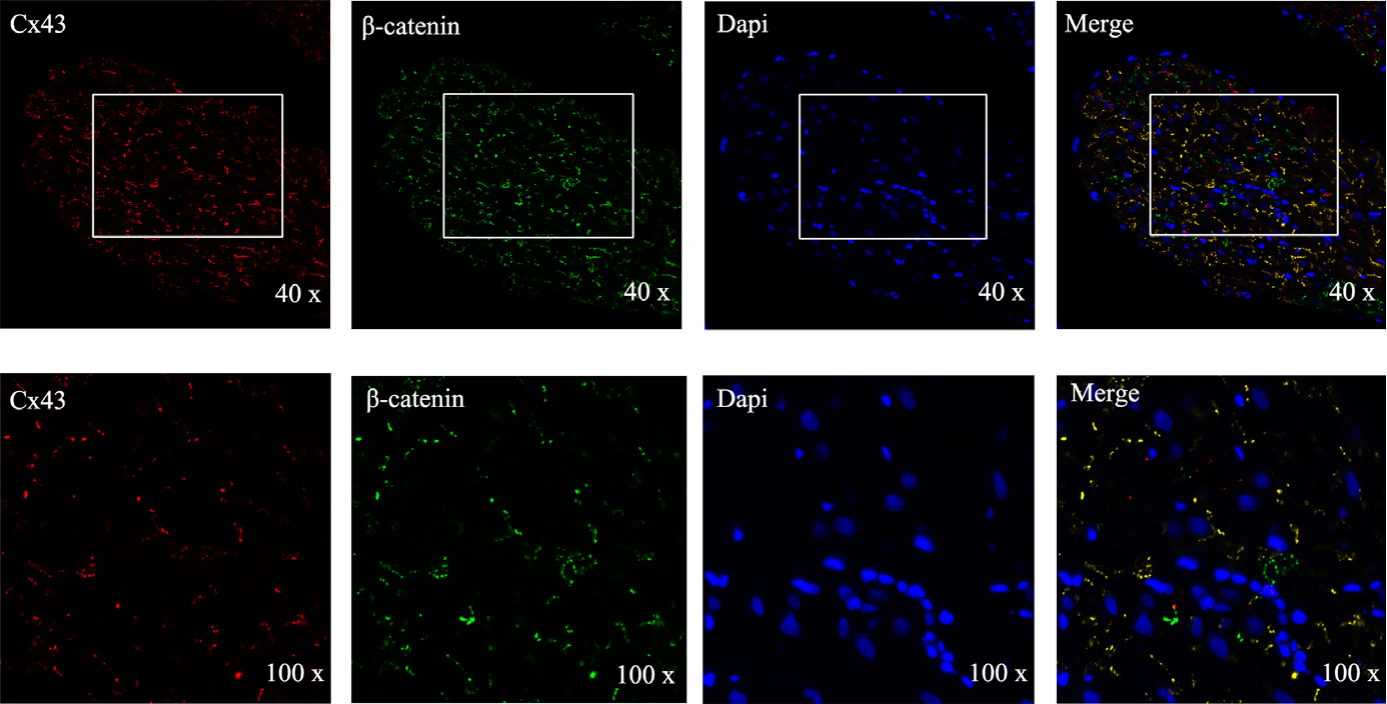
Double immunofluorescent staining of Cx43 (red) and β‐catenin (green) antibodies (intercalated disk associated protein) in cardiac sympathetic denervation (CSD) group. The colocalizations of Cx43 with β‐catenin (yellow) were observed at the membrane in cardiomyocytes (original magnification ×40/100). Cx43 indicates connexin 43.

To observe the expression of Cx43 in the right atria, western blot was performed in the 4 groups. Figure 7A shows bands corresponding to total Cx43, with quantitative analyses presented in Figure 7B. Compared with the sham group, total C43 expression in CIH rats was decreased. Compared with the sham and CIH groups, the expression of Cx43 in CSD and CIH+CSD groups was gradually increased by western blot. Immunohistochemistry was performed to determine the cellular localization of Cx43 in right atria tissue, with anti‐Cx43 mouse monoclonal antibody. Figure 8A presents images of immunohistochemical staining of Cx43, which was reduced in CIH rats and increased in CSD treatment rats, and quantitatively similar to the western blot date. Double labeling immunofluorescent staining confirmed the decrease in Cx43 after CIH exposure, and CSD treatment increased Cx43 expression (Figure 8C). These data indicated that the Cx43 expression changed by CIH exposure or CSD treatment, and the alteration of Cx43 may play a key point in the genesis of CIH induced AF. In addition, to determine which structure of the cardiomyocytes expressed Cx43, double labeling immunofluorescent staining was performed with the following cell‐specific markers: β‐catenin antibody (intercalated disk associated protein, Sigma, 1:150), DAPI (a marker of nucleus; blue). The colocalizations of Cx43 with β‐catenin was observed at the membrane in cardiomyocytes (Figure 9).

## Discussion

Characteristics of OSA are repetitive episodes of partial (hypopnea) or complete (apnea) obstruction of the upper airway during the sleep cycle.^1^ Using an experimental animal model in the basic study of OSA may contribute to a better understanding of its pathophysiological mechanisms and cardiovascular consequences. The most frequently used model is chronic intermittent hypoxia (CIH), which repeats repeated hypoxia events during sleep by exposing experimental animals to the hypoxia/reoxygenation component of OSA.^7^, ^11^ In addition, CIH is a distinct pathological mechanism of OSA, which is recognized as an independent risk factor for cardiovascular diseases.^10^ Ideal animals for OSA models should be relatively inexpensive, manipulable, and unrestricted and have small, easily blocked upper airways.^7^ CIH models allow the animals to be exposed to hypoxic conditions for long periods that can last several months, allowing the exploration of chronic outcomes that develop in human disease. In this study, we mimicked OSA with a CIH rat model that presents the characteristics of OSA, and is convenient for investigating the pathophysiology of OSA and finding appropriate treatments.

OSA has been consistently considered a cardiovascular risk factor, and its treatment has long been observed in association with arrhythmia.^21^ Despite the lack of rigorous, randomized controlled trials, there are plenty of data showing that not only does untreated OSA provide the substrates and triggers for AF but that OSA, itself, is also a therapeutic target for the management of cardiovascular disease in the general population and of AF in particular.^22^ The profound role of OSA has grown into a focal point in the pathogenesis and treatment of AF. OSA and AF are both common, often under diagnosed conditions with serious sequelae. An association between OAS and AF has been recognized but the nature of this relationship remains a topic of active debate. OSA can increase the inducibility of AF and shorten the atrial effective refractory period, which are potent triggers for AF.^23^, ^24^ OSA and AF share many risk factors and complications, including sex, age, hypertension, coronary artery disease, and congestive heart failure.^25^ In addition, there are many different potential mechanisms by which OSA is related to the occurrence of AF. Several potential pathophysiological mechanisms, including intermittent hypoxemia, intrathoracic pressure shifts, sympathovagal imbalance, and upregulation of inflammation led to atrial arrhythmogenesis.^2^ Autonomic nerve imbalance has been regarded as a crucial factor for OSA to improve AF.^14^, ^26^ Sympathetic neural tone is linked to cardiovascular disease and is increased in OSA patients during both sleep and wakefulness and correlates with OSA severity.^22^

Several studies indicate that the autonomic nervous system plays a critical role in the occurrence and maintenance of human AF.^24^ OSA induces repeated hypoxia, which leads to chemoreflex and increased sympathetic activity, leading to BP elevation and tachycardia and the end of tachycardia, especially at the end of the apnoeic episodes.^27^ In sustained AF of both animals and humans, significant nerve sprouting and sympathetic hyperinnervation were observed.^15^ Previous studies have shown that ganglionated plexi ablation significantly inhibited OSA‐induced AF inducibility and atrial effective refractory period shortening.^28^ Renal sympathetic denervation is likely to affect cardiac electrophysiology by relieving the hyperactivity of the sympathetic nervous system, and it inhibits OSA‐induced AF.^17^, ^24^ Similar to these studies, we found that CSD has the potential to reduce postapneic BP increases and AF susceptibility after CIH. In addition, we also observed that CIH induced sympathetic nerve hyperinnervation and CSD treatment reduced sympathetic innervation. These findings implied that autonomic dysregulation in CIH can serve to trigger AF, but CSD can inhibit AF inducibility; a possible mechanism is CIH‐induced, AF‐associated sympathovagal imbalance.

In addition, OSA causes LA stretch, and repeated stretch is the main cause of atrial remodeling tied to AF.^29^ Previous studies have demonstrated that AF causes atrial remodeling, which has a key role in the progression and perpetuation of the arrhythmia.^30^ Atrial remodeling can be found in terms of electrical and structural changes.^31^ Structural remodeling includes atrial enlargement, fibrosis, hypertrophy, myolysis, and other degenerative changes.^29^ Electrical remodeling includes reduced myocardial potassium and calcium channels and remodeled gap junction connexin hemichannels, manifesting as changes to atrial myocardial effective refractory periods and conduction velocities.^32^ The reverse is also true. Atrial structural and electrical remodeling play a key role in the pathogenesis of AF. They lead to anatomic and electrical heterogeneity rendering the atria prone to re‐entry circuits and sustained AF.^32^ Atrial structural remodeling are considered to be 2 core processes of atrial fibrosis and enlargement.^31^ A considerable body of date have demonstrated that P‐wave duration and dispersion are useful and non‐invasive markers linked to paroxysmal AF. Patients with paroxysmal AF were reported to have consistently prolonged P‐wave duration and dispersion.^30^ In our study, we demonstrated that CIH caused a significant increase in left atrial diameter, right atrial diameter, P‐wave duration and inducibility and that CSD attenuated the increase of left atrial diameter, right atrial diameter, P‐wave inducibility, and duration. These findings agree with published results upholding a role for atrial structural and electrical heterogeneity. Moreover, it seems that CSD decreases cardiac SA and central nerve drive, leading to decreased atrial electrical and structural heterogeneity.

Intercellular communication is known to play a crucial role in electrical conduction velocity in heart tissue through gap junctions.^33^ Gap junctions are composed of a family of proteins called connexins, which mediate the transmission of action potential between cells, coordinate the electrical and mechanical activity of the heart and ensure that the heart is simultaneously electromechanical.^34^ Cx43 is known as the major gap junction protein that regulates the intercellular transport of small molecules and is involved in inducing AF and stimulating the sympathetic nerve.^35^ The expression of Cx43 was slowly and reversibly reduced by hypoxia itself.^36^ Cx43 expression was investigated among 4 groups in this study to better comprehend that mechanism in OSA induced AF. We found that total Cx43 expression in CIH rats was decreased and that the Cx43 expression was gradually increased after CSD treatment. In addition, we also observed that CIH can easily induce AF occurrence. Furthermore, it was observed in our study that in the condition of CIH, decreased cardiac sympathetic activity could reduce AF easily. The alternation of Cx43 is 1 of the possible mechanisms resulting in this phenomenon. These results suggest that CSD treatment increased the Cx43 expression, and the alteration of Cx43 may play a key point in the genesis of CIH‐induced AF.

## Conclusions

To the best of our knowledge, our study is the first to investigate the potential interplay among CIH, AF, and CSD in animal models. First, CIH induced atrial remodeling and increased AF inducibility. Second, CSD treatment reduced postapneic BP increases and AF susceptibility, which could attenuate CIH associated structural atrial arrhythmogenic remodeling. Then, CIH induced sympathetic nerve hyperinnervation, and CSD treatment reduced sympathetic innervation, which may have affected CIH‐induced, AF‐associated sympathovagal imbalance. Furthermore, the Cx43 expression changed through CIH exposure or CSD treatment, and the alteration of Cx43 may play a key role in the genesis of CIH‐induced AF.

### Limitations

Despite our compelling and novel findings, the study had limitations. First, this study was conducted in rat models and has some limitations, as do all animal models of human disease. Second, we assessed atrial dilation or electrical remodeling in a CIH rat model of OSA; additional studies focusing on atrial fibrosis or cardiomyocyte apoptosis are warranted. Third, we performed complete surgery of CSD and examined the density of cardiac sympathetic nerves in this study. However, precise measurements of SA during and after obstructive events deserve further study.

## Disclosures

None.

## Supporting information


**Figure S1.** Blood pressure values at the end study among the 4 groups.
**Figure S2.** Arterial blood gases during conditioning procedure shows significant oxygen desaturation and hypoventilation in each group.
**Figure S3.** Echocardiographic average values of LAD, RAD, LVPWd, LVPWs, LVIDd, LVIDs, EF, FS, and LV mass in each group.
**Figure S4.** The statistical values of RR interval, HR, P wave duration, and T amplitude among 4 groups.
