# Supplementary material for: Cardiac Sympathetic Denervation Suppresses Atrial Fibrillation and Blood Pressure in a Chronic Intermittent Hypoxia Rat Model of Obstructive Sleep Apnea
Source: J Am Heart Assoc. 2019 Feb 13;8(4):e010254. doi: 10.1161/JAHA.118.010254 (PMC6405657; doi:10.1161/JAHA.118.010254)
Supplement: Supplementary file 1 — Figure S1. Blood pressure values at the end study among the 4 groups. Figure S2. Arterial blood gases during conditioning procedure shows significant oxygen desaturation and hypoventilation in each group. Figure S3. Echocardiographic average values of LAD, RAD, LVPWd, LVPWs, LVIDd, LVIDs, EF, FS, and LV mass in each group. Figure S4. The statistical values of RR interval, HR, P wave duration, and T amplitude among 4 groups. [file JAH3-8-e010254-s001.pdf]

Supplemental Material

Figure S1. Blood pressure values at the end study among the four groups.

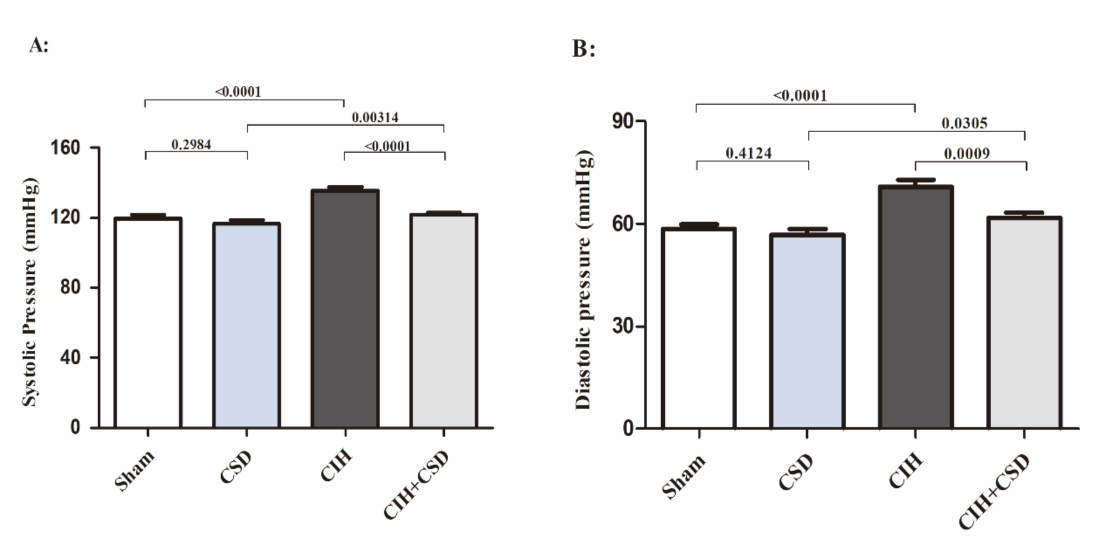

A: The systolic BP of the four groups. B: The diastolic BP in each group. Sham: sham group; CSD: cardiac sympathetic denervation group; CIH: chronic intermittent hypoxia group; CIH+CSD: chronic intermittent hypoxia with cardiac sympathetic denervation group; BP: blood pressure.

Figure S2. Arterial blood gases during conditioning procedure shows significant oxygen desaturation and hypoventilation in each group.

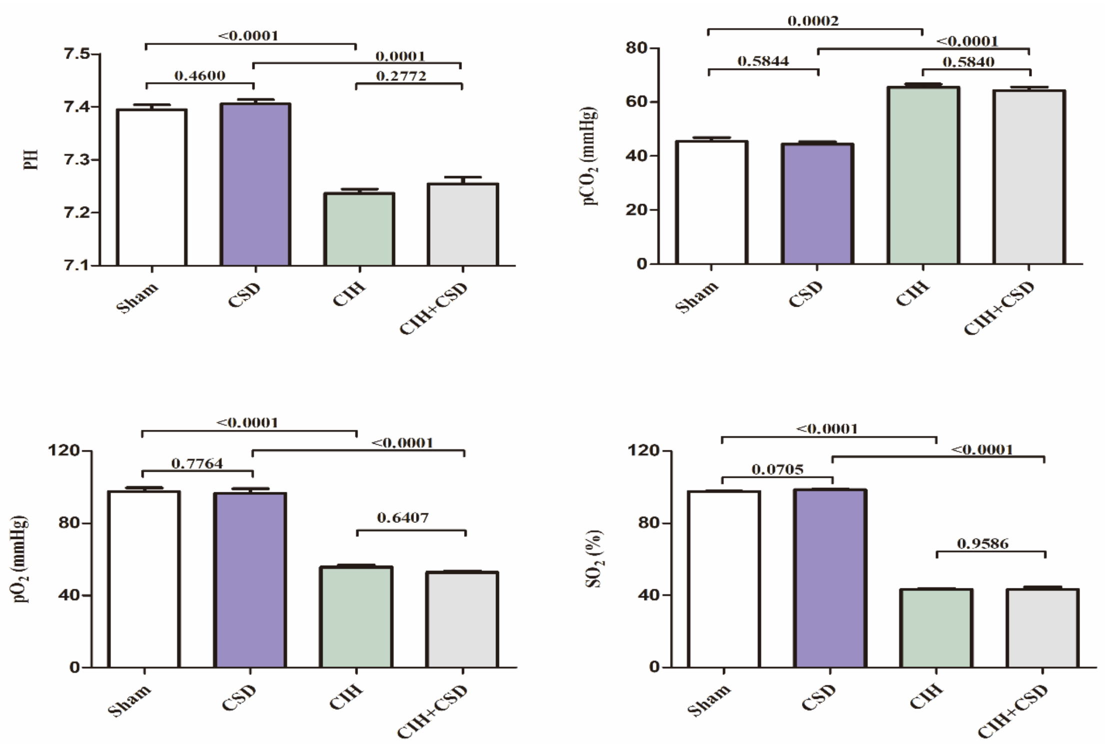

Sham: sham group; CSD: cardiac sympathetic denervation group; CIH: chronic intermittent hypoxia group; CIH+CSD: chronic intermittent hypoxia with cardiac sympathetic denervation group.

Figure S3. Echocardiographic average values of LAD, RAD, LVPWd, LVPWs, LVIDd, LVIDs, EF, FS, and LV mass in each group.

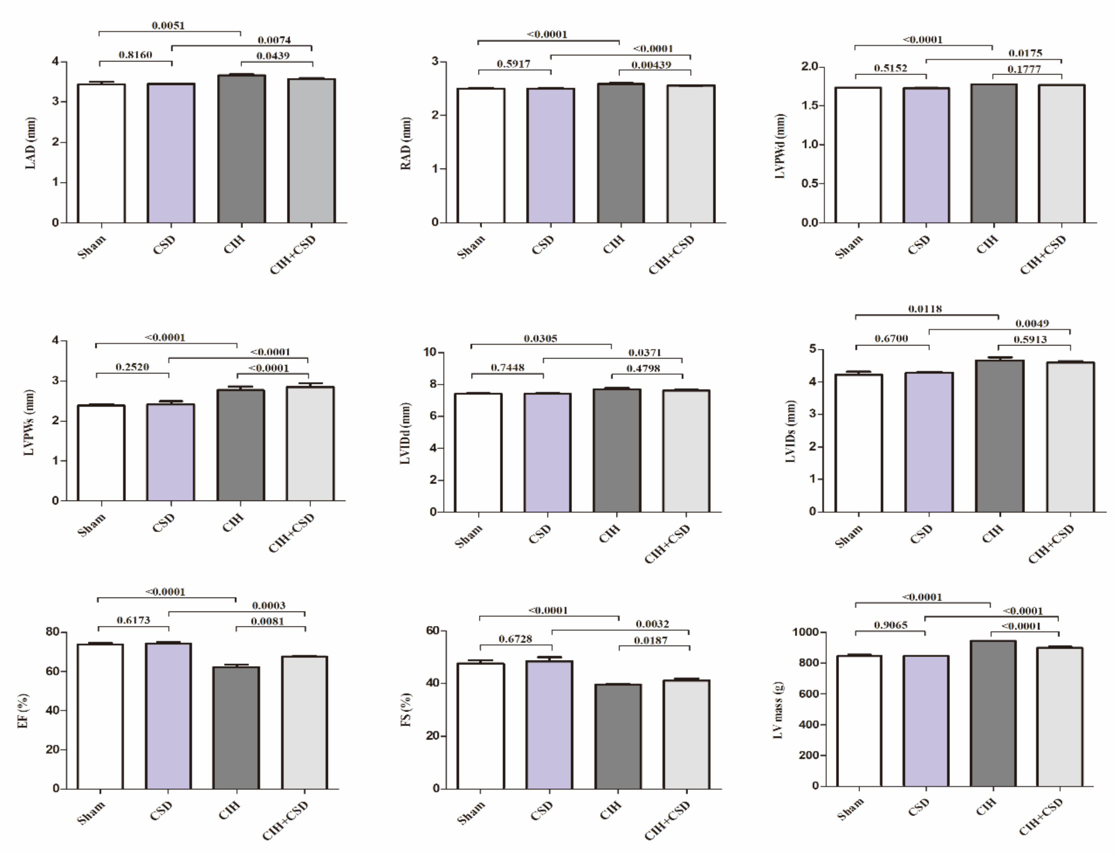

Sham: sham group; CSD: cardiac sympathetic denervation group; CIH: chronic intermittent hypoxia group; CIH+CSD: chronic intermittent hypoxia with cardiac sympathetic denervation group; LAD: left atrial diameter; RAD: right atrial diameter; LVIDd: left ventricular internal diameter in diastole; LVIDs: left ventricular internal diameter in systole; LVPWd: the left ventricle posterior wall in diastole; LVPWs: the left ventricle posterior wall in systole; EF: ejection fraction; FS: fractional shortening; LV mass: left ventricular mass.

Figure S4. The statistical values of RR interval, HR, P wave duration, and T amplitude among four groups.

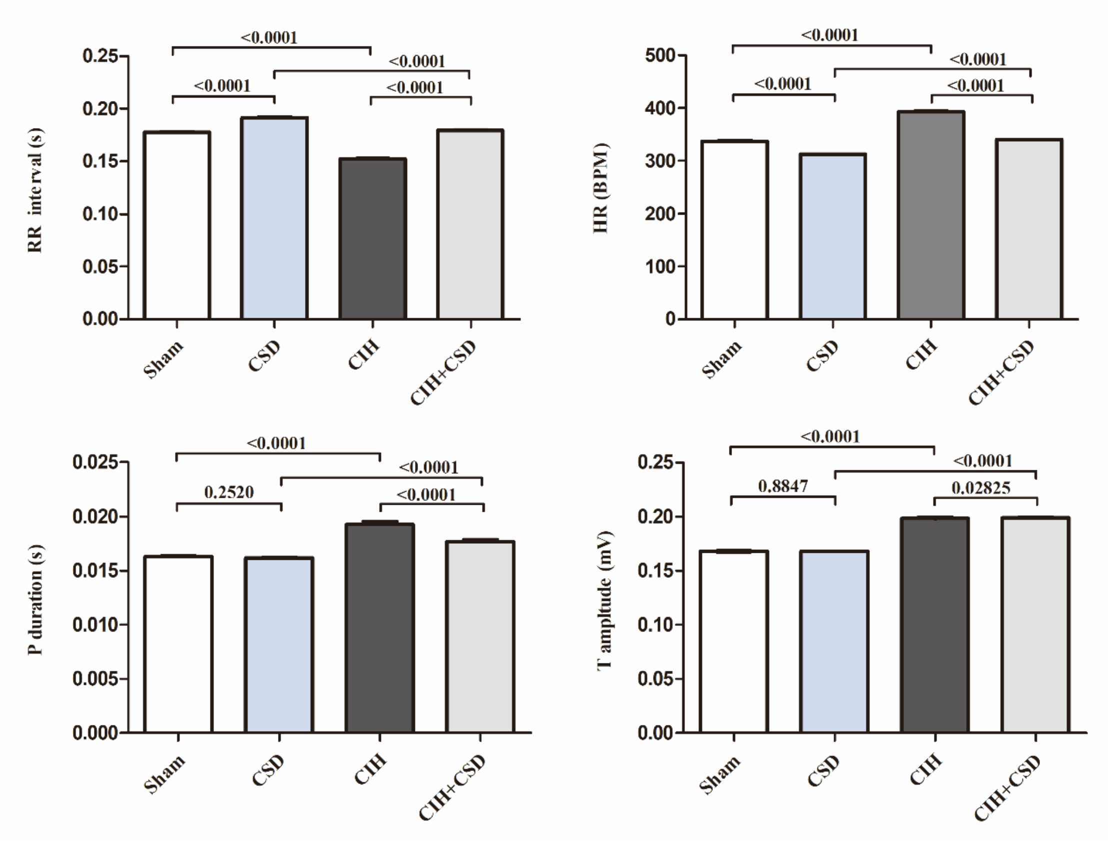

Sham: sham group; CSD: cardiac sympathetic denervation group; CIH: chronic intermittent hypoxia group; CIH+CSD: chronic intermittent hypoxia with cardiac sympathetic denervation group; HR: heart rate.
